# Isolation and Phylogenetic Analysis of Atypical Porcine Pestivirus Isolates Identified in Russian Swine Herds

**DOI:** 10.3390/v17010002

**Published:** 2024-12-24

**Authors:** Afshona Anoyatbekova, Anton Yuzhakov

**Affiliations:** Federal State Budget Scientific Institution “Federal Scientific Center VIEV”, 109428 Moscow, Russia; anton_oskol@mail.ru

**Keywords:** atypical porcine pestivirus, genome, pestivirus, isolate, cell culture, phylogenetic analysis

## Abstract

Atypical porcine pestivirus (APPV) was first identified in 2015 in North America by high-throughput sequencing. APPV is associated with congenital tremor A-II and is widely distributed worldwide. In this study, a total of 2630 samples of domestic pigs obtained from 14 regions of Russia from 2020 to 2024 were screened for APPV presence by qRT-PCR. APPV was detected in 12 farms located in eight regions. The overall positive rate was 8.8%. It has been established that APPV has been circulating in Russian swine herds since at least 2020. The phylogenetic analysis demonstrated that the Russian isolates are variable and assigned into three clusters. The isolates from the Krasnoyarsk Krai, Belgorod, Tomsk, and Kursk regions and the Republic of Buryatia share a high nucleotide identity (94.3–98.8%) with the Hungarian strains, while the isolates from the Moscow and Pskov regions share a nucleotide identity (89.2–94.3%) with strains from the USA. The isolate from the Republic of Mordovia has a high nucleotide identity (97.1%) with the South Korean strain. *In vitro* studies of the Russian isolates revealed the replication of the Belgorod 151 strain in SPEV cells. Thus, this is the first large-scale study that confirms the circulation of APPV in swine herds in Russia and describes its isolation in cell culture.

## 1. Introduction

The genus *Pestivirus* within the *Flaviviridae* family includes several economically significant viruses such as classical swine fever virus (CSFV), bovine viral diarrhea virus (BVDV), and border disease virus (BDV), which are responsible for severe disease in swine, cattle, and small ruminants [1,2]. The high genetic diversity and the continued expansion of members of the *Pestivirus* genus constitute a worldwide concern. According to the latest taxonomy update by the International Committee on Taxonomy of Viruses (ICTV), the *Pestivirus* genus currently comprises 19 different species [2].

*Pestiviruses* are enveloped viruses with a single-stranded positive-sense RNA genome approximately 11.3–13.0 kb in length. The RNA genome contains a single open reading frame (ORF) flanked by a 5′- and 3′-untranslated region (UTR). The ORF encodes a polyprotein that is processed into four structural proteins (Core, Erns, E1, and E2) and eight nonstructural proteins (Npro, p7, NS2, NS3, NS4A, NS4B, NS5A, and NS5B) [2,3]. All species of the genus *Pestivirus* share a similar genomic organization except for *Phococena pestivirus*, which lacks the Npro gene [4].

Over the past two decades, three novel pestiviruses have been identified in pigs, causing different forms of the disease: Linda virus, Bungowannah virus, and atypical porcine pestivirus (APPV) [5,6,7,8]. APPV (*Pestivirus scrofae)* was first discovered in 2015 in North America by metagenomic sequencing in swine serum samples [8].

Later studies indicated that APPV is associated with congenital tremor (CT) type A-II and splay leg (SL) in piglets [9,10,11]. CT is a neurological disorder that occurs in newborn piglets and is characterized by head and body tremors as a result of hypomyelination of the central nervous system lasting from several weeks to months [11]. Splay leg is characterized by the impairment of the adducting muscles of the hind limbs and sometimes of the forelegs [11]. The clinical manifestations of APPV in infected piglets are usually mild; nevertheless, they may range from asymptomatic to clinically severe [8,9,10,12,13,14]. In adult pigs, APPV infection is usually subclinical [8,9,10].

APPV seems to be restricted to domestic pigs and wild boars. The occurrence of APPV infection in domestic pigs has been confirmed in the Netherlands [13], Canada [14,15], various states throughout the USA [8,9,16], Austria [17], China [18,19], Spain [20], South Korea [21], Brazil [22], Great Britain [23,24], Taiwan [24], Hungary [25], Japan [26], Italy [27], Serbia [24], Sweden [28], Switzerland [29], Denmark [30], and Germany [10,31], and in wild boars in Sweden, South Korea, Italy, Spain, the USA, and Germany [27,28,32,33]. Although APPV was discovered recently, some retrospective studies indicate that the virus was likely circulating in pig populations for decades prior to the initial reports [20,25,29]. Due to the increased detection of APPV in the global pig population, there is an urgent need to explore those countries where the virus has not been previously studied.

Thus, in this study, APPV circulation in the swine population in Russia was determined and the overall virus detection rate was estimated. Moreover, APPV isolation in cell cultures was described.

## 2. Materials and Methods

### 2.1. Sample Collection

All of the samples used in this study were obtained by qualified personnel in the framework of routine diagnostic surveillance on farms. The samples were obtained from 24 pig farms located in 14 regions of the Russian Federation. All of the investigated farms use the farrow-to-finish production system. From the Republic of Buryatia, the Republic of Mordovia, and the Novosibirsk, Vologda, Kursk, Tyumen, Belgorod, Pskov, and Sverdlovsk regions, samples were obtained from nine farms—one farm per region. From the Krasnoyarsk Krai and Tomsk regions, samples were collected from four pig farms per region, and from the Kemerovo and Kirov regions, samples were collected from two pig farms per region. From the Moscow region, samples were obtained from three pig farms. Altogether, 2630 samples collected in the period of 2020–2024 were investigated, including serum (*n* = 2420), tissue specimens (lungs, lymph nodes, testes, and spleen) (*n* = 143), feces (*n* = 53), bronchoalveolar lavage (BAL) (*n* = 4), and semen (*n* = 10). The numbers of samples per region and farm are given in Table 1 and Supplementary Table S1. Information about the breeds and health status of the pigs was not available. All of the serum samples were taken from random pigs of various production age groups (suckling piglets [*n* = 149], weaned pigs [*n* = 426], sows [*n* = 519], and fatteners [*n* = 1326]), per the accompanying documentation. Data regarding the age of the pigs for other clinical and pathological materials were unknown.

### 2.2. Sample Processing and RNA Extraction

The serum samples were pooled by five prior to nucleic acid extraction. The individual serum samples from the positive pool were then subjected to RNA extraction. A piece of tissue from each organ sample with 5 mL of phosphate-buffered saline (PBS) (Syntol, Moscow, Russia) was homogenized in a 50 mL sterile centrifuge tube and centrifuged at 3000× *g* for 10 min. The feces samples were suspended in PBS, vortexed, and centrifuged at 2500× *g* for 15 min. The semen samples were diluted to 10% in PBS before RNA extraction. The bronchoalveolar lavage (BAL) samples were centrifuged at 3000× *g* for 15 min. The supernatants of the centrifuged samples were then collected and used for nucleic acid extraction. The tissues, feces, BAL, and semen were tested individually.

The total RNA was extracted from 100 µL samples with the commercial kit “Riboprep” (FBIS Central Research Institute of Epidemiology of Rospotrebnadzor, Moscow, Russia) following the instructions of the manufacturer. The extracted nucleic acids were directly used for qRT-PCR analysis or stored at −70 °C until use.

### 2.3. Quantitative Real-Time Polymerase Chain Reaction (qRT-PCR)

For diagnostic purposes, we used primers and a probe targeting the 5′ UTR described by Kauffman C. et al. (2019) [29]. qRT-PCR for APPV detection was carried out using the one-step RT-PCR kit (AlphaFerment, Moscow, Russia) following the manufacturer’s instructions. The qPCR reaction was performed on BioRad CFX-96 (BioRad, Hercules, CA, USA) with the following conditions: 1 cycle at 50 °C for 30 min and 95 °C for 5 min (RT step), followed by 45 cycles at 95 °C for 15 s, 58 °C for 15 s, and 72 °C for 20 s, whereby the first five cycles were carried out without plate reading. The samples with Ct (cycle threshold) values ≤ 35 were considered positive.

### 2.4. Reverse Transcriptase PCR and Sequencing

The reverse transcription and polymerase chain reaction were conducted separately. The synthesis of cDNA was carried out with a commercial reagent kit, “RT-1”, for RT-PCR (Syntol, Moscow, Russia) according to the manufacturer’s instructions using Random 6 primer (Syntol, Moscow, Russia). For the PCR, primers APPV_4273-fw and APPV_5169-rev targeting the NS2-NS3 regions of APPV were used [10]. The PCR was performed in a 25 µL reaction volume consisting of 2.5 µL of 10× Turbo buffer (Evrogen, Moscow, Russia), 0.5 µL of dNTPs mix (New England Biolabs, Ipswich, MA, USA), 16.25 µL of nuclease-free water, 0.25 µL of Hot Start Taq Polymerase (Evrogen, Moscow, Russia), and 1.5 µL of 10 pmol of each primer (forward and reverse) and 2.5 µL of cDNA. The amplifications were performed in a MiniAmp (Thermo Fisher Scientific, Waltham, MA, USA) thermocycler using the following thermal profile: denaturation at 94 °C for 5 min; 38 cycles of 95 °C for 20 s, 56 °C for 20 s, and 72 °C for 40 s; and a final extension step at 72 °C for 5 min. The size (896 bp) of the PCR products was analyzed by 1% agarose gel electrophoresis containing Tris-acetate buffer solution (pH 8.0) and ethidium bromide (0.5 µg/mL) using 2 µL of the PCR product. The remaining amount of PCR product in a volume of 23 µL was purified by the Monarch PCR&DNA Cleanup Kit (New England Biolabs, Ipswich, MA, USA) according to the manufacturer’s instructions. The purified DNA was sequenced in both directions by the Big Dye 3.1 Terminator Cycle Sequencing Kit (Thermo Fisher Scientific, Carlsbad, CA, USA) following the manufacturer’s instructions and carried out on the ABI PRISM 3130 Genetic Analyzer (Thermo Fisher Scientific, Carlsbad, CA, USA) sequencing device.

### 2.5. Phylogenetic Analysis

The obtained sequences were analyzed and assembled into a final consensus using the SeqMan Lasergene 11.1.0 (DNASTAR, Madison, WI, USA). The phylogenetic analysis was carried out by the Molecular Evolutionary Genetics Analysis version 7.0 (MEGA 7.0) software. The obtained sequences were aligned by the MUSCLE algorithm. The phylogenetic tree was constructed using the maximum likelihood (ML) method based on the general time reversible (GTR), (G + I) model. The topology evaluation was performed by 1000 bootstrap replications. The pairwise genetic distances were calculated according to the Tamura 3-parameter model.

### 2.6. Cell Lines

Porcine embryo kidney cell line (SPEV), swine kidney (SK), swine testis (ST), African green monkey kidney cells (Vero), subclone of the African green monkey kidney MA104 (MARC-145), immortalized pig spleen cells (SIPS), and lamb testes (LT) from “Specialized collection of continuous, somatic cell cultures of domestic and wild animals at the Federal Scientific Centre VIEV” (FSC VIEV Cell Collection, Moscow, Russia) were used for the APPV isolation. The SK, LT, and ST were maintained in Eagle’s minimum essential medium (PanEco, Moscow, Russia) with 10% bovine serum (BS) (Biosera, Cholet, France), 10 units/mL penicillin, and 10 μg/mL streptomycin (PanEco, Moscow, Russia). The MARC-145, Vero, and SIPS were grown in Dulbecco’s modified Eagle’s medium with high glucose (PanEco, Moscow, Russia) supplemented with 10% fetal bovine serum (FBS) (IntlKang, Beiling, China), 2 mM L-glutamine, and 10 U/mL of penicillin and 10 μg/mL of streptomycin (PanEco, Moscow, Russia). The cell lines were cultured in 25 cm^2^ tissue culture flasks for three to four days post-seeding at 37 °C. The 0.25% Trypsin-EDTA (PanEco, Moscow, Russia) was used for the cell’s dissociation from the flasks. Prior to infection, all of the cell lines were confirmed to be free of BVDV, CSFV, BDV, APPV, and *Mycoplasmas.*

### 2.7. Virus Isolation

For the virus isolation, the cells were seeded in 48-well tissue culture plates (Nest, Wuxi, China) in a 1 × 10^6^ cells/mL concentration and kept at 37 °C in a 5% CO_2_ incubator. The positive serum samples with the lowest Ct values were used for the APPV isolation. The cell culture monolayers were infected when they were approximately 70% confluent. The serum samples were sterilized through a 0.45 μM syringe filter (Jet BioFil, Guangzhou, China) to remove bacteria and fungi, and then a 0.1 mL volume was used for cell inoculation. For mock infection, Eagle medium was used. After adsorption for 2 h at 37 °C, a maintenance medium (0.5 mL per well) containing 2% of growth serum was added without removing the viral inoculum, and the cultures were incubated at 37 °C with 5% CO_2_ for five to six days. After a single freeze–thaw cycle (−70 °C and +8 °C) followed by centrifugation at 2000× *g* for 15 min, the supernatants were harvested and used for the subsequent passage. The APPV replication in the cell cultures was monitored by qRT-PCR. Three blind APPV passages were performed before the sample was discarded as negative.

### 2.8. Statistical Analysis

The database was constructed using Excel (Microsoft Corporation, Redmond, Washington, DC, USA). The APPV detection rate was calculated as a ratio of the number of positive samples to the total number of tested samples (number of positives/total tested) × 100%. The statistical analysis was conducted with Past 4.17 software. Fisher’s exact test was used to compare the detection rate of APPV in the different diagnostic specimens and age groups. The results with a *p*-value of < 0.05 (*) were considered statistically significant.

## 3. Results

### 3.1. APPV Detection Rates in Swine Herds in Russia

A total of 2630 samples were tested for APPV presence, and 232 (8.8%) of them were positive by qRT-PCR (Table 1 and Table S1). The virus genome was detected in 12 out of 24 tested pig farms (50%). We considered a farm positive even if one positive sample was detected. The APPV occurrence ranged from 1.6% to 25.3% in the positive farms.

**Figure 1:**
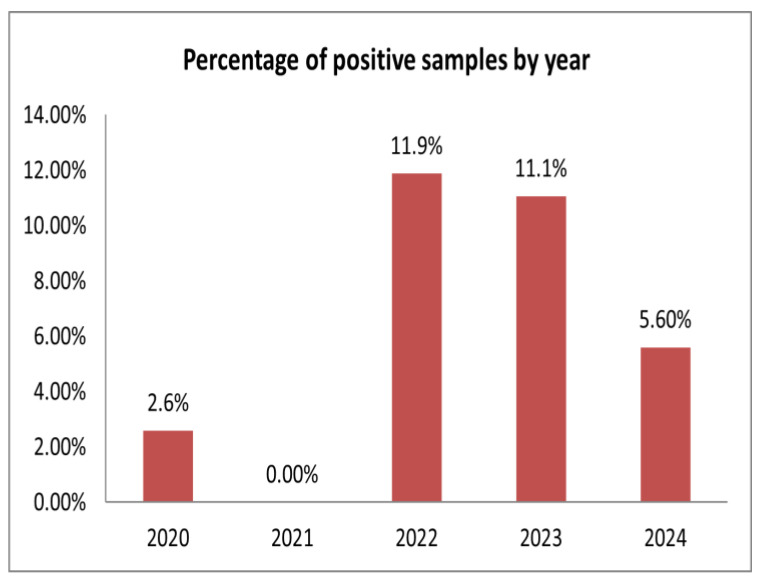
The annual APPV detection rates in swine herds. A total of 2630 clinical and pathological materials collected from 24 pig farms in 14 geographical locations in Russia were tested by qRT-PCR, and the results were grouped by year. The *X*-axis represents the 2020–2024 years, while the *Y*-axis demonstrates the percentage of positive samples.

Krasnoyarsk Krai (KK) accounts for the majority of tested samples, where the overall APPV detection rate was 7.3% (62/853). In all KK farms (KKA–KKD), APPV was detected despite the fact that varying numbers of samples were obtained in different years. The positive rates of KKA, KKB, KKC, and KKD were 8.2% (20/244), 7.7% (18/235), 10.2% (13/127), and 4.5% (11/247), respectively. Only two farms (TB and TD) were APPV-positive out of the four tested in the Tomsk region. The percentage of positive samples was 5% (2/40) and 2.9% (2/70), respectively. In the Moscow region, the overall percentage of APPV-positive samples was estimated at 10.9% (13/119). MC (18.6%) was the single farm positive for APPV in this region. Among all of the samples tested, the samples from the farm in the Belgorod region had the highest positive ratio of 25.3% (100/396), followed by the Kursk (2/12) and Pskov regions’ farms (23/138), which amounted to 16.7%. In the farms of the Republic of Buryatia and Republic of Mordovia, APPV was identified in 3.1% (14/455) and 8.8% (14/160) of the tested samples, respectively. The APPV genome was not detected in the samples obtained from the pig farms in the Vologda, Kemerovo, Kirov, Tyumen, Sverdlovsk, and Novosibirsk regions. To explore the temporal distribution of APPV, all of the samples from the farms in different geographical locations were further analyzed by year. In 2020, we obtained samples only from the farms in Krasnoyarsk Krai and detected three positive cases in the set of 116 investigated, corresponding to a 2.6% detection rate (Table 1, Figure 1).

All of the examined samples (*n* = 282) in 2021 were APPV-negative. A high frequency of APPV identification was observed in 2022 and comprised 11.9% (152/1280). In 2023, a total of 434 samples were analyzed, of which 11.1% (*n* = 48) were positive. In 2024, the APPV-positive rate was 5.6% (29/518).

#### APPV Detection Rates in Specimen Types and Different Age Groups

The highest APPV detection frequency was observed in the serum samples and tissue specimens (lungs, testis, liver, lymph nodes) at 9% and 10.4%, respectively (Table 2 and Table S1).

In total, 217 out of 2420 serum samples and 15 out of 143 tissue specimens were APPV-positive. No statistical differences (*p* > 0.05) were found in terms of the APPV detection rates between the serum samples and tissues. Mean Ct values of between 13.39 and 25.27 were found for serum samples and between 16.06 and 19.11 for tissue specimens. All of the feces, BAL, and semen samples obtained from the same farms turned out to be negative.

**Figure 2:**
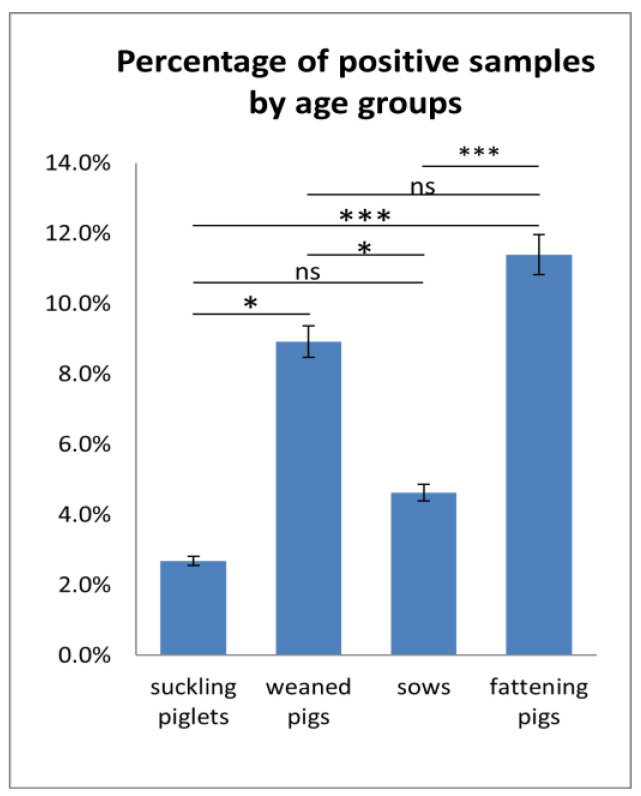
The percentage of APPV-positive samples in different age groups (%). Statistically significant differences were assessed by Fisher’s exact test. The asterisks above the columns represent the statistically significant differences (ns *p* > 0.05, * *p* < 0.05; *** *p* < 0.0001).

The serum samples collected during 2020–2024 spanned four age groups: suckling piglets, weaning pigs, sows, and fattening pigs. Cross-sectional surveillance using serum allowed us to establish the APPV detection rate in all age groups of pigs. The fattening pigs were the group with the highest APPV detection rate (Figure 2). The APPV genome was found in 11.4% (151/1326) of all tested samples. The APPV-positive rate in the weaned piglets and sows was 8.9% (38/426) and 4.6% (24/519), respectively. The suckling piglets were the group with the lowest APPV detection rate. The virus was found in 2.7% (4/149) of all tested samples.

Statistically significant differences (*p* < 0.05) were detected between the weaned pigs and the suckling piglets and between the weaned pigs and the sows in terms of the APPV detection rate (Figure 2). Between the suckling piglets and sows, as well as between the weaned pigs and fattening pigs, the frequency of APPV detection was statistically insignificant (*p* > 0.05). When examining the APPV detection rate between the suckling piglets and the fattening pigs and between the sows and the fattening pigs, the differences were statistically significant (*p* < 0.001).

### 3.2. Phylogenetic Analysis and Nucleotide Identity of APPV

**Figure 3:**
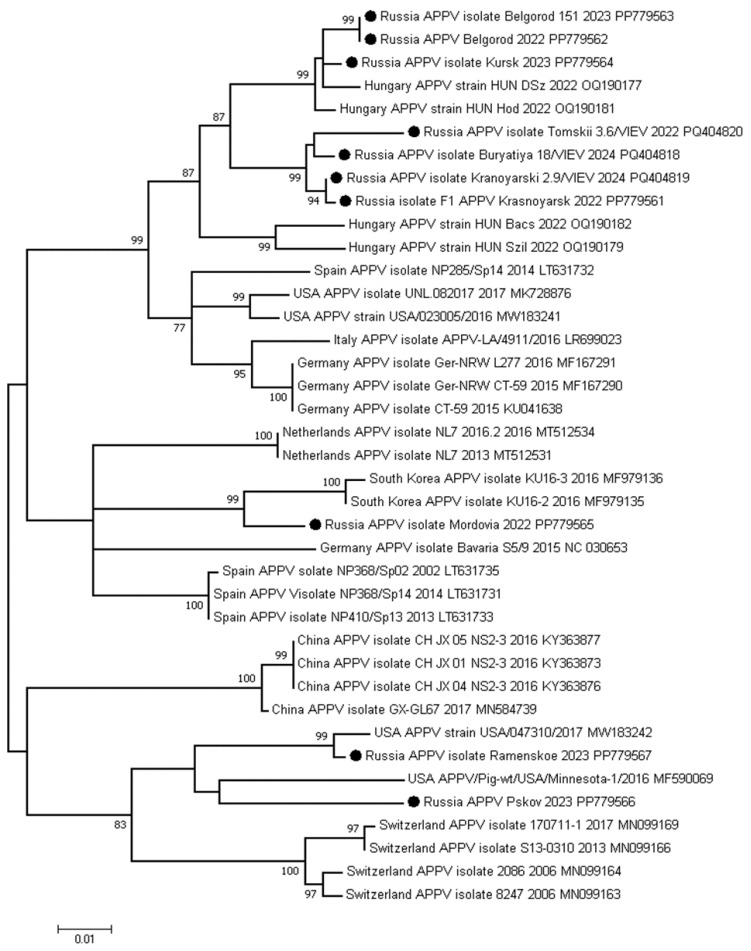
The phylogenetic tree of a partial NS2-NS3 gene of the APPV strains from GenBank and Russian isolates. The dendrogram was constructed by the ML method and the GTR model (G + I). Bootstrap support values (≥70) are provided. The scale bar indicates 0.01 expected changes per site per branch. The APPV sequences obtained in the current study are indicated by a black circle (●).

For the phylogenetic analysis, 10 partial NS2-NS3 sequences were obtained from the positive farms in eight regions using Sanger sequencing. The nucleotide sequence identity between the samples from different Russian geographical locations varied from 86.4% to 100% and from 85.6% to 100% with APPV strains from GenBank. Based on the phylogenetic analysis, the Russian isolates were divided into three separate clusters (Figure 3).

The first cluster included isolates from the Belgorod (PP779563, PP779562), Kursk (PP779564), and Tomsk (PQ404820) regions, Krasnoyarsk Krai (PQ404819, PP779561), and the Republic of Buryatia, which are closely related to the strains isolated in Hungary in 2022. The nucleotide sequence identities within the clusters ranged from 95.0% to 98.8% (OQ190181) and from 94.3% to 98.6% (OQ190177), respectively. The detailed data on genetic distances are presented in Table 3.

The Mordovia strain (PP779565) showed a nucleotide identity (97.1%) with the strains from South Korea (MF979135, MF979136) and formed a second cluster. The isolates from the Moscow and Pskov regions are grouped in the third cluster together with the strains isolated in the USA in 2017. The Ramenskoye isolate (PP779567) from the Moscow region shares a nucleotide identity with the USA047310 (MW183242) strain (92.4%) and APPV/Pig-wt/USA/Minnesota-1/2016 (MF590069) (94.3%). The Pskov isolate has a 93.6% nucleotide sequence identity with APPV/Pig-wt/USA/Minnesota-1/2016 (MF590069). Nucleotide diversity was not found among the Russian isolates collected in different years and at different farms.

### 3.3. APPV Isolation in Cell Cultures

**Figure 4:**
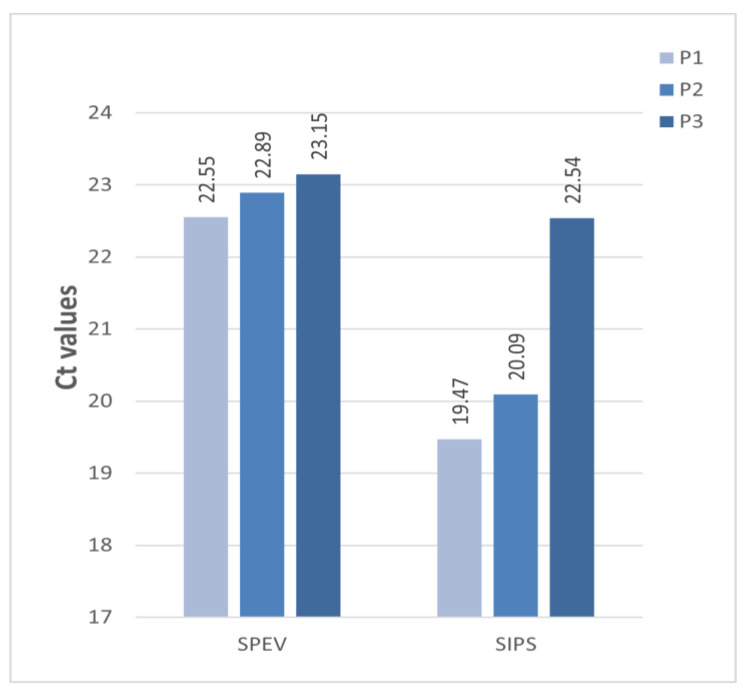
Replication of the Belgorod 151 strain in SPEV and SIPS cell lines during consecutive three passages. The Ct values of each passage are indicated.

To isolate the APPV from the field material, the susceptibility of various cell cultures was evaluated. Serum samples from each APPV-positive region that had the lowest Ct values were subjected to virus isolation. In each cell culture, three blind passages of the virus were performed. The collection of starting inoculums for each subsequent passage was monitored after 5 five days post-infection by qRT-PCR. Throughout all three passages, the cytopathic effect of the virus was not observed. The qRT-PCR results showed that the isolates had different abilities to replicate *in vitro.* APPV was detected only in the supernatant of the SPEV and SIPS cells infected with the Belgorod 151 isolates. The infections with other isolates were negative. None of the isolates replicated in the ST, SK, MARC-145, and LT cell lines. A detailed study of the 151 Belgorod replication in the SPEV and SIPS cell cultures revealed a slight increase of Ct with each passage (Figure 4).

The mean Ct value in the SPEV cell line after the third passage was 23.15, and in SIPS, 22.54, given that the initial Ct of the sample was 19.00. No virus was detected, in either of the mock-infected cells throughout the isolation attempt. SIPS cells might be as suitable for APPV isolation as SPEV cells, depending on the amount of virus load in the sample. All of the attempts to propagate the APPV from other regions in the cell lines were unsuccessful.

## 4. Discussion

Over the past few years, next-generation sequencing has demonstrated capabilities for the discovery of emerging viruses in the livestock industry. Using this exact method, APPV was detected in serum samples by Hause et al. in 2015 [9]. The pathogenicity of the virus was determined through the experimental infection of dams during gestation [9]. It was uncovered that APPV causes congenital tremors type A-II in newborn piglets [9,10]. Soon afterwards, numerous studies reported APPV infection in swine herds, highlighting its prevalence globally [10,13,14,15,16,17,18,20,22,23,25,26,27,29,30,31,33,34,35].

In this study, we intended to detect the APPV genome in different diagnostic materials and assess the virus detection rates in 24 pig farms located in 14 regions of Russia. All of the samples were obtained in the framework of routine diagnostic surveillance, so the numbers of samples per farm and region varied. Altogether, 2630 samples from domestic pigs collected in 2020–2024 were tested for APPV by qRT-PCR.

The overall detection rates in Russian swine herds were found to be 8.8% (232/2630), which is almost consistent with the prevalence rate of APPV in European countries, at 8.9% (130/1460) [24]. In Italy, the highest detection rate of the APPV genome was 17.5% (35/200) [24]. In Switzerland, 13% of APPV cases were positive [29]. In Germany, the antibody-positive rate for APPV was 16.3% (182/1115) [31]. Over a three-year period, the prevalence of APPV in the Midwest of the United States was 19.00% (339/1785) [36]. In China, the total APPV-genome-positive rate from 2017 to 2021 was 7.08% (69/975) [35]. Our results demonstrated temporal changes in the APPV detection rates from 2.6% to 5.6% for the entire study period (2020–2024). However, in 2022and 2023, the APPV-positive rate in the tested pig farms was 11.9% and 11.1%, respectively, despite the fact that there were no positive samples detected in 2021. The temporal changes in the APPV detection rate can be explained by the collection of different amounts of samples from pigs of various age groups and from farms in different geographical locations. It has been confirmed that APPV has been circulating in the territory of the Russian Federation since at least 2020. A retrospective study of cerebellum samples from Germany and Hungary confirmed the presence of APPV in CT-affected piglets over a decade ago [10,25]. In Switzerland, samples of slaughtered pigs collected in 1986 were confirmed to be APPV-positive [29]. In Spain, APPV was found in samples collected in 1997 [20]. These findings emphasize the necessity of conducting a retrospective analysis to determine the time point of APPV introduction into the swine population in Russia.

The results of the analysis of the samples obtained from the 24 pig farms demonstrated that APPV was circulating in 12 pig farms (50%) located in eight regions of Russia. It should be noted that the APPV detection rates on farms notably varied. In the farms in the central and northwestern areas of the European part of Russia, a higher virus detection rate was observed. Thus, the highest APPV-positive rate was determined in a farm from the Belgorod region (25.3%), followed by the farms from the Pskov and Kursk regions (16.7%). It should be mentioned that Russia’s main industrial pig farming industry is concentrated in these regions, thus the possibility of virus shedding might increase. However, since only one pig farm in these areas was tested, we are unable to estimate the APPV prevalence throughout the region. In the pig farms in the Asian part of Russia, lower APPV detection rates were noticed. Even though the largest number of samples was obtained from the four pig farms in Krasnoyarsk Krai, the percentage of positive samples was low at 7.3% (62/853). The lowest APPV detection rates were observed in samples obtained from the farms in the Tomsk region and the farm in the Republic of Buryatia: 3.1% and 1.6%, respectively. It is worth noting that a relatively small number of samples was obtained from farms in some regions, and the number of farms was limited throughout the entire study period. Although our study was intended to estimate the overall APPV detection rate, it cannot provide a precise estimation at the regional level. This would have required a larger number of swine herds in each region.

To identify the appropriate specimens for APPV detection, various diagnostic materials collected from the same farms were tested. According to Gatto et al. (2017), the cerebellum is the most consistently positive sample type from CT piglets and could represent a target for APPV replication [37]. However, apart from the cerebellum and lymph nodes, APPV has been detected in almost all of the organs, the feces, and the serum of CT piglets [8,9,10,17,27]. The main APPV replication sites in tissues are still unknown, as the pathogenesis during APPV infection has not been sufficiently studied. In the United States, APPV was detected in the semen, preputial swabs, and preputial fluids of commercial boars, suggesting that transiently infected boars without a clinical history of CT could shed APPV via semen [16].

In our study, all of the investigated semen, BAL, and feces samples were negative for APPV. All of the tested serum samples were categorized into four age groups based on an analysis of the accompanying documentation: sows, sucking piglets, weaners, and fattening pigs. As no data were available for the tissues and other clinical materials, they were not included in the cross-sectional analysis. The APPV detection rate among the suckling piglets was 2.7% (4/149) (*p* < 0.001), which is lower compared to other age groups. The frequency of APPV detection in the weaned pigs was 8.9% (38/426). According to Cagatay et al. (2019), piglets infected horizontally through contact with persistently infected pigs have viremia for several weeks and eventually develop an active immune response to APPV infection [38]. The sows tested positive for APPV in 4.6% (24/519) of cases. Although APPV was found in animals of all age groups, the fattening pigs were the age group with a significantly (*p* < 0.05) higher detection rate (11.4%). These findings are consistent with Kauffmann et al. (2019), who also observed a high prevalence (13%) of APPV among fattening pigs in Switzerland [29]. According to several studies, adult pigs remain asymptomatic during APPV infection but shed the virus [9,12,27,29,30,35]. It is assumed that during the reorganization of infected fattening pigs into separate groups, there is a risk of transient infections from carrier animals [29].

From each investigated region, we sequenced at least one APPV-positive sample for phylogenetic analysis based on the NS2-NS3 gene. The Russian isolates were grouped into three clusters and shared a high nucleotide identity with the strains from Hungary, the USA, and South Korea. It is known that one of the most significant characteristics of the APPV genome is its high genetic variability. Yuan et al. (2021), based on the NS5a gene, assigned three major clades (clade I–III) for APPV [39]. Consequently, clade I includes all of the isolates found in the United States and Europe, and several isolates from China. Clades II and III comprise only isolates from China [39]. These findings suggest that various APPV clades might be present in a single area. Furthermore, we sequenced two isolates from the Belgorod Region farm that were discovered in different years, as well as from from two farms in Krasnoyarsk Krai (KKB and KKD) that were found in separate years. Nevertheless, no nucleotide diversity was discovered between them. All of the farms we studied are from different geographical areas, and many of them are located at a considerable distance from each other. It remains unknown whether pigs from the investigated areas were traded or moved between farms or imported from other countries. As a result, we are unable to predict or estimate the routes of infection. Thereby, it is necessary to conduct a more in-depth study of the epidemiological process and mechanisms of infection transmission.

As virus isolation is generally considered to be the gold standard for diagnosing pestiviruses, we attempted to isolate the APPV in cell cultures. The cell lines of different species origins (SK, SPEV ST, SIPS, MARC-145, Vero, LT) were examined for susceptibility. Since tissue samples were not obtained from every APPV-positive region, the serum samples with the best Ct values were used for the virus isolation in cell cultures. Apart from the Belgorod 151 isolate, replication of the other isolates was not recorded in any of the studied cultures. These findings are in line with studies that reported the inability of various APPV isolates to replicate *in vitro* [8,17,23]. Successful virus isolation typically depends on both the virus load in the sample and the cell culture system. Shiokawa et al. (2023) reported that SK-L cells are suitable for the isolation and artificial production of APPV [40]. The replication of Belgorod 151 was observed in the SPEV and SIPS cell lines. According to some studies, SPEV cells might be the most sensitive cell line for APPV isolation [41,42]. Thus, further passages are required to confirm the long-term cultivation of Belgorod strains in SPEV and SIPS cells and to study the biological properties of the virus *in vitro.*

## 5. Conclusions

To the best of our knowledge, this is the first report on the detection rate, phylogenetic analysis, and isolation of APPV in the Russian Federation. Our results confirmed that APPV is common among pigs in Russia, but significant differences in APPV detection rates between farms, age groups, and clinical and pathological materials were observed. The phylogenetic analysis revealed high nucleotide sequence identities with the APPV strains available from GenBank. APPV isolation in cell cutures will allow for the study of the pathogenecity of the virus in pigs and the development of diagnostic tools for the rapid detection and accurate diagnosis of infection. The circulation of APPV in swine herds in Russia indicates the necessity to monitor the spread of APPV using systematic surveillance.

## Figures and Tables

**Table 1:** The total number of samples obtained from pig farms in Russian regions and the percentage of APPV-positive samples.

Investigated Area	Farm’s ID	2020		2021		2022		2023		2024		Total by Farm/Region	
		Positive/Total Tested	Positive %	Positive/Total Tested	Positive %	Positive/Total Tested	Positive %	Positive/Total Tested	Positive %	Positive/Total Tested	Positive %	Positive/Total Tested	Positive %
Krasnoyarsk Krai (Farms/Total)	KKA	0/27	0%	0/20	0%	13/114	11.4%	0/21	0%	7/62	11.3%	20/244	8.2%
	KKB	1/34	2.9%	0/20	0%	11/138	7.9%			6/43	13.9%	18/235	7.7%
	KKC	1/19	5.3%	0/9	0%	7/64	10.9%			5/35	14.3%	13/127	10.2%
	KKD	1/36	2.8%	0/20	0%	9/107	8.4%	0/8	0%	1/76	1.3%	11/247	4.5%
	Total	3/116	2.6%	0/69	0%	40/423	9.5%	0/29	0%	19/216	8.8%	62/853	7.3%
Republic of Buryatia	RB			0/67	0%	0/143	0%	8/125	6.4%	6/120	5%	14/455	3.1%
Tomsk Region (Farms/Total)	TA					0/42	0%			0/30	0%	0/72	0%
	TB					1/10	10%			1/30	3.3%	2/40	5%
	TC					0/39	0%			0/28	0%	0/67	0%
	TD					0/40	0%			2/30	6.7%	2/70	2.9%
	Total					1/131	0.8%			3/118	2.5%	4/249	1.6%
Vologda Region	VR			0/48	0%							0/48	0%
Kemerovo Region (Farms/Total)	KA							0/10	0%	0/19	0%	0/29	0%
	KB									0/16	0%	0/16	0%
	Total							0/10	0%	0/35	0%	0/45	0%
Kirov Region (Farms/Total	KRA					0/19	0%					0/19	0%
	KRB					0/11	0%					0/11	0%
	Total					0/30	0%					0/30	0%
Tyumen Region	TR									0/15	0%	0/15	0%
Belgorod Region	BR			0/50	0%	97/332	29.2%	3/14	21.4%			100/396	25.3%
Kursk Region	KR							2/12	16.7%			2/12	16.7%
Republic of Mordovia	RM					14/160	8.8%					14/160	8.8%
Moscow Region (Farms/Total)	MA			0/44	0%							0/44	0%
	MB					0/5	0%					0/5	0%
	MC							12/56	21.4%	1/14	7.1%	13/70	18.6%
	Total			0/44	0%	0/5	0%	12/56	21.4%	1/14	7.1%	13/119	10.9%
Novosibirsk Region	NR			0/4	0%			0/23	0%			0/27	0%
Pskov Region	PR							23/138	16.7%			23/138	16.7%
Sverdlovsk Region	SR					0/56	0%	0/27	0%			0/83	0%
Total by year	12/24 (50%)	3/116	2.6%	0/282	0%	152/1280	11.9%	48/434	11.1%	29/518	5.6%	232/2630	8.8%

**Table 2:** The total APPV detection rates in specimen types from all tested farms.

Specimen Type	Total Investigated	Number of Positive	%
Serum	2420	217	9%
Semen	10	0	0%
Feces	53	0	0%
BAL	4	0	0%
Tissue specimens	143	15	10.5%
Total	2630	232	8.8%

**Table 3:** The nucleotide sequence identities (%) among the APPV strains from GenBank and the Russian isolates obtained in this study.

Russian Isolates	APPV Strains from GenBank				
	Hungary OQ190181	Hungary OQ190177	USA MW183242	USA MF590069	South Korea MF979135
Belgorod_2022_	98.8	98.6	93.5	85.9	89.6
Belgorod 151 2023	98.8	98.6	93.5	85.9	89.6
Tomskii_3.6/VIEV_2022	95.0	94.3	88.4	84.6	87.9
Kranoyarski_2.9/VIEV_2024	96.5	95.9	94.5	85.9	88.8
F1_APPV_Krasnoyarsk_2022	96.3	95.7	89.3	86.3	88.5
Ramenskoe_2023	90.3	89.5	92.4	94.3	89.8
Pskov_2023	88.6	88.7	89.2	93.6	87.5
Mordovia_2022	91.0	91.2	90.5	89.9	97.1
Kursk_2023	99.1	98.9	93.9	86.2	90.1
Buryatiya_18/VIEV_2024	96.1	95.5	94.3	85.7	88.5

## Data Availability

The data presented in the study are deposited in the NCBI GenBank repository. The accession numbers are PQ404818-PQ404820 and PP779561–PP779567.

## Supplementary Materials

The following supporting information can be downloaded at: https://www.mdpi.com/article/10.3390/v17010002/s1. Table S1.
