# Supplementary material for: Isolation and Phylogenetic Analysis of Atypical Porcine Pestivirus Isolates Identified in Russian Swine Herds
Source: Viruses. 2024 Dec 24;17(1):2. doi: 10.3390/v17010002 (PMC11768848; doi:10.3390/v17010002)
Supplement: Supplementary file 1 [file viruses-3315881-supplementary.pdf › viruses-3315881-supplementary.pdf]

Regions	Farms	Type of Samples										Total of samples by regions	
		Serum		Tissues		Feces		BAL		Semen			
		Positive/ total	Positive %	Positive/ total	Positive %	Positive/ total	Positive %	Positive/ total	Positive %	Positive/ total	Positive %	Positive/ total	Positive %
**Krasnoyarski Krai**	KKA	20/204	9.8%	0/19	0%	0/21	0					20/244	8.2%
	KKB	18/211	8.5%	0/16	0%	0/8	0					18/235	7.7%
	KKC	13/127	10.2%									13/127	10.2%
	KKD	11/247	4.5%									11/247	4.5%
**Republic of Buryatia**	RB	14/455	3.1%									14/455	3.1%
**Tomsk Region**	TA	0/72	0%									0/72	0%
	TB	2/40	5%									2/40	5%
	TC	0/67	0%									0/67	0%
	TD	2/70	2.9%									2/70	2.9%
**Vologda Region**	VR	0/48	0%									0/48	0%
**Kemerovo Region**	KA	0/6	0%	0/10	0%	0/3	0%					0/19	0%
	KB	0/10	0%			0/16	0%					0/26	0%
**Kirov Region**	KRA	0/19	0%									0/19	0%
	KRB	0/11	0%									0/11	0%
**Tyumen Region**	TR	0/15	0%									0/15	0%
**Belgorod Region**	BR	97/382	25.4%	3/14	21.4%							100/396	25.3%
**Kursk Region**	KR			2/12	16.7%							2/12	16.7%
**Republic of Mordovia**	RM	14/160	8.8%									14/160	8.8%
**Moscow Region**	MA	0/44	0%									0/44	0%
	MB	0/5	0%									0/5	0%
	MC	3/10	30%	10/50	20%					0/10	0%	13/70	18.6%
**Novosibirsk Region**	NR	0/23	0%					0/4				0/27	0%
**Pskov Region**	PR	23/138	16.7%									23/138	16.7%
**Sverdlovsk Region**	SR	0/56	0%	0/22	0%	0/5	0%					0/83	0%
**Total by regions**	24	217/2420	9%	15/143	10.5%	0/53	0%	0/4	0%	0/10	0%	232/2630	8.8%
